# Genetic HLA Study of Kurds in Iraq, Iran and Tbilisi (Caucasus, Georgia): Relatedness and Medical Implications

**DOI:** 10.1371/journal.pone.0169929

**Published:** 2017-01-23

**Authors:** Antonio Arnaiz-Villena, Jose Palacio-Grüber, Ester Muñiz, Cristina Campos, Javier Alonso-Rubio, Eduardo Gomez-Casado, Shadallah Fareq Salih, Manuel Martin-Villa, Rawand Al-Qadi

**Affiliations:** 1 Department of Immunology, University Complutense, School of Medicine, Madrid Regional Blood Center, Madrid, Spain; 2 Department of Inmunología Animal, Instituto Nacional de Investigación y Tecnología Agraria y Alimentaria (INIA), Autopista A6, Hipódromo, Madrid, Spain; 3 HLA Typing Department, Dohuk Specialized Laboratory Center, Dohuk, Iraq; National Cheng Kung University, TAIWAN

## Abstract

Kurds from Iraq (Dohuk and Erbil Area, North Iraq) have been analyzed for HLA genes. Their HLA genetic profile has been compared with that of other Kurd groups from Iran and Tbilisi (Georgia, Caucasus) and also Worldwide populations. A total of 7,746 HLA chromosomes have been used. Genetic distances, NJ dendrograms and correspondence analyses have been carried out. Haplotype HLA-B*52—DRB1*15 is present in all three analyzed Kurd populations. HLA-A*02-B*51-DRB1*11 is present in Iraq and Georgia Kurds. Haplotypes common to Iran and Iraq Kurds are HLA DRB1*11—DQB1*03, HLA DRB1*03—DQB1*02 and others in a lower frequency. Our HLA study conclusions are that Kurds most probably belong to an ancient Mediterranean / Middle East / Caucasian genetic substratum and that present results and those previously obtained by us in Kurds may be useful for Medicine in future Kurd transplantation programs, HLA Epidemiology (HLA linked diseases) and Pharmacogenomics (HLA-associated drug side effects) and also for Anthropology. It is discussed that one of the most ancient Kurd ancestor groups is in Hurrians (2,000 years BC).

## Introduction

HLA is the most polymorphic genetic system described in man. It contains several linked *loci* which encode for cell surface proteins that have an important function in activating immune response after antigenic presentation. New allele variants are frequently being described (i.e.: 1,883 *HLA-DRB1* alleles have been recorded by June 2016) [1]. HLA gene frequencies have both a large degree of variability among populations and a striking geographical correlation. These frequencies are useful to infer genetic background and ethnical constitution of modern human groups and also for inferring migrations of ancient ones [2]. In addition, certain combinations of contiguous alleles between HLA neighboring *loci* show a characteristic frequency due to the robust linkage disequilibrium among them or are distinctive in many extant populations [3]. Also, HLA allele frequencies are unique for studying the origins of relatively homogeneous groups, like the Kurd people living in Iraq. Other HLA gene characteristics are their link to disease and to different responses to drug treatments in patients according to different HLA alleles. Certain HLA alleles affect drug response to treatment in about sixteen different diseases including AIDS [4]. This is important for personalized drug treatment design (including ethnic groups with specific certain high allele frequencies), particularly if other already obtained Kurd HLA results are also included (from Georgia and Iran, in present study) and samples are further increased.

**Fig 1:**
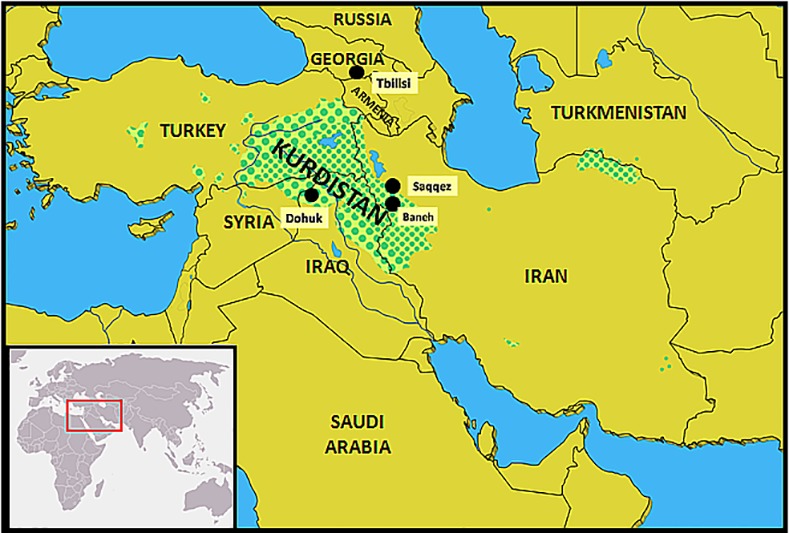
Geographical location of Duhok in the Kurd Autonomous Province of Iraq. Erbil is province capital and is located about 170 km South East Duhok. (This Fig is similar but not identical to Fig 1 in Ref [13]; It has been included only for illustrative purposes).

On the other hand, Kurd people live in different countries in the Near East such as Syria, Armenia, Turkmenistan, Kazakhstan, Turkey, Iraq and Iran, the so called Kurdistan ("land of Kurds") (Fig 1, Table 1). Kurdistan is a region placed South Caucasus and North of ancient Mesopotamia. According to genetic studies (like HLA) in Turkish and Kurdish populations, a Anatolian-Mediterranean source for both populations was put forwards; it may be possible that Kurds are initially coming from ancient Hurrians, reviewed in [5,6]. Studies performed with mtDNA and Y-chr have also been done for Kurds, however there is no firm conclusion to infer that most Kurd people have originated either from Middle East and/or from Central Asia [7,8]. Most probably, Kurd people gene pool majority may be composed of an admixture of North Mesopotamian (Caucasus) and Near East peoples; Central Asia gene input is not discarded [5,9,10,11,12,13]. Kurds have mainly been defined by their ancestry, language and cultural uses. Estimations of Kurds number are nowadays between 23 to 41 million people; see Table 1 for numbers and country distribution.

**Table 1:** Kurds population around the World [14,15].

Kurds Population					
Kurdistan			Kurds Diaspora		
Country	Number of inhabitants (x10^3^)	Country	Number of Inhabitants (x10^3^)	Country	Number of Inhabitants (x10^3^)
Turkey	12,000–22,500	Germany	800	Switzerland	35
Iran	3,350–8,000	France	150	Denmark	30
Iraq	4,000–6,500	Israel	100–200	Jordan	30
Syria	2,000–2,500	Sweden	83.6	Austria	23
Armenia	37.5	Belgium	80	Greece	22
Georgia	14	Netherlands	70	USA	15.4
Azerbaijan	6.1	Russia	63.8	Kyrgyzstan	13.2
		UK	50	Canada	11.7
		Kazakhstan	42.3	Finland	10.7
Total of Kurds:	23,038,000–41,300,000				

In the present paper, a population of Kurds living in North Iraq (Dohuk and Erbil area, North Mosul, Fig 1) has been studied in order to: 1) Determine the HLA class I (A, B and C) and class II (DRB1 and DQB1) allelic Kurd lineages (hereafter “alleles” for simplicity) and specific HLA haplotypes by using standard DNA based techniques, 2) Compare Iraq Kurd HLA profile with those of Central Asia, Siberian, Mediterranean and other World ethnic groups (Table 2) with specific computer programs in order to find out bases of HLA and disease linkage and origins of Kurd people using genetic distances comparisons, Neighbour Joining (NJ) trees and correspondence analyses, 3) obtaining Kurd HLA profile that may be used for preventive HLA Pharmacogenomics and a virtual regional future transplant waiting list among population, and finally 4) Kurd HLA profiles from Tbilisi (Georgia-Caucasus), Iran and Iraq are also compared among themselves.

**Table 2:** Populations used for this study.

Population	N	Reference	Population	N	Reference
Algerians	106	[27]	Lebanese	59	[21]
Armenian	141	[19]	Macedonians	178	[38]
Ashkenazi Jews	80	[28]	Mansi	68	[31]
Baloch	100	[29]	Moroccans	98	[39]
Berbers (Souss)	98	[30]	Moroccan Jews	113	[40]
Buryat	25	[31]	Negidal	35	[31]
Chuvash	82	[32]	Non-Ashkenazi Jews	80	[28]
Cretans	144	[33]	Palestinians	165	[41]
Croatians	105	[21]	Russians	200	[42]
Evenks	35	[34]	Sardinians	91	[19]
French	321	[19]	Spaniards	88	[43]
Germans	295	[19]	Spanish Basques	83	[43]
Georgians	119	[35]	Svan	80	[44]
Gorgan	69	[36]	Todja	22	[31]
Zoroastrians	65	[37]	Tofalar	43	[31]
FarsParsi	73	[37]	Tuvinians	197	[3]
Italians	284	[19]	Iraq Kurds	209	*Present study*
Japanese	493	[19]	Iran Kurds	60	[13]
Kets	22	[34]	Georgia Kurds	30	[5]
			Ulchi	73	[31]

## Material and Methods

### Population sample

209 healthy unrelated blood donor volunteers from the cities of Dohuk and Erbil and their area, North Iraq (Fig 1) were class I and class II typed. Unrelatedness and other sample parameters were checked by Drs. R. Al-Qadi and Shadallah Fareq Salih. Erbil is the capital of Iraq Kurdish Autonomous Region placed 175 km southeast Dohuk. The city of Dohuk is located in Kurdistan region in the North of Iraq, 70 km North Mosul and 60 km far from both Sirian and Turkish borders (36°51′00″N 42°59′00″E); Dohuk is historically and continuously inhabited by Kurds since 1000 BC. Dr. Rawand Al-Qadi established at Dohuk General Specialized Laboratory Center has taken samples. Written consent to participate in the present study was signed by each individual. University Complutense Ethics Review Board Committee reviewed and approved this study which was subsequently funded by Ministry of Health and Economy (see Acknowledgments).

All subjects in the study and their grandparents were born in the same area. We compared our data with those of worldwide populations (see Table 2), obtaining genetic distances, relatedness trees and correspondence analyses. Comparisons were done with 7,746 HLA chromosomes.

### HLA genotyping

110 Iraq Kurd samples were genotyped for HLA-A, -B, -C, -DRB1 and -DQB1 using Lifecodes HLA-SSO kit, following manufacturer’s suggestions (Immucor Transplant Diagnostic, Inc. Stamford, Connecticut, USA). The rest of the samples (n = 92) were typed using Polymorphism Chain Reaction-Sequence Specific Primers (PCR-SSP) method already mentioned [16]; other methods are now currently used [2].

### Statistical analysis

Statistical analysis was done with Arlequin v2.0 software [17,18,19]. Frequent complete extended HLA haplotypes were obtained from: 1) HLA loci haplotype frequencies [18,19]; 2) described haplotypes present in other populations [18,19]; and 3) HLA haplotypes if they appeared in more individuals and also the second haplotype was not undefined [18,19]. Reference tables of the 11^th^ and 12^th^ International HLA Workshops were used for comparing phenotype and haplotype frequencies [20,21].

Phylogenetic trees were obtained with the allelic frequencies as described [22,23], using DISPAN programs [24,25]. Correspondence analysis was carried out as described [26]; it displays a general view of the relationships among populations.

HLA allele typing data from Iran Kurds [13] have been converted to low resolution typing data in order to be able to carry out all analyses with as many populations as possible, while relatedness resolution is satisfactory given the very polymorphic HLA system [1].

## Results

### HLA allele frequencies found in Kurd Iraq population: comparison with other Populations

The expected and observed allele frequency values for HLA-A, -B, -C, -DRB1 and -DQB1 shows that the population is in Hardy-Weinberg equilibrium. Table 3 depicts HLA allele frequencies found in the sampled population. Sixteen different HLA-A, twenty-seven different HLA-B and thirteen different HLA-C alleles were founds in class I. Only seven HLA-A alleles, nine HLA-B alleles and seven HLA-C alleles had frequencies higher than 4% (-A*01, -A*02, -A*03, -A*11, -A*24, -A*26, -A*32, -B*07, -B*08, -B*18, -B*35, -B*38, -B*41, -B*44, -B*51, -B*52, -C*04, -C*06, -C*07, -C*12, -C*15, -C*16 and -C*17). Twelve different HLA-DRB1 alleles and five different HLA-DQB1 alleles were found. Only eight HLA-DRB1 alleles and four HLA-DQB1 alleles had frequencies higher than 4% (-DRB1*01, -DRB1*03, -DRB1*04, -DRB1*07, -DRB1*11, -DRB1*13, -DRB1*14, -DRB1*15, -DQB1*02, -DQB1*03, -DQB1*05 and -DQB1*06).

**Table 3:** HLA-A, -B, -C, -DRB1, and -DQB1 allele frequencies in Iraq Kurds population.

Allele	Allele Frequencies %	Allele	Allele Frequencies %	Allele	Allele Frequencies %
**HLA-A**		35	15.55	07	19.14
01	13.16	37	0.48	08	1.91
02	16.75	38	4.31	12	16.51
03	15.31	40	2.63	14	6.94
11	9.57	41	6.46	15	8.13
23	1.67	44	10.29	16	9.81
24	13.88	45	0.72	17	4.78
26	6.94	47	0.24		
29	2.15	48	0.24	**HLA-DRB1**	
30	3.83	49	3.35	01	4.31
31	2.15	50	1.91	03	15.07
32	5.50	51	15.55	04	12.68
33	3.83	52	5.98	07	7.89
66	0.48	53	1.20	08	1.67
68	3.83	55	2.87	09	1.20
69	0.24	57	1.20	10	1.91
80	0.48	58	1.44	11	26.08
		67	0.24	13	9.09
**HLA-B**		73	0.48	14	6.46
07	4.07	81	0.24	15	11.00
08	7.18	**HLA-C**		16	2.63
13	1.20	01	3.11	**HLA-DQB1**	
14	2.15	02	2.15	02	22.25
15	2.87	03	3.59	03	42.58
18	5.26	04	15.79	04	0.96
27	1.67	05	2.87	05	17.46
		06	5.02	06	16.75

DRB1 alleles were used to compare our three Kurd samples with other populations in NJ analysis. It was not possible to perform this study with HLA class I allele frequencies due to the lack of class I studies in many worldwide available populations (Table 2).

**Fig 2:**
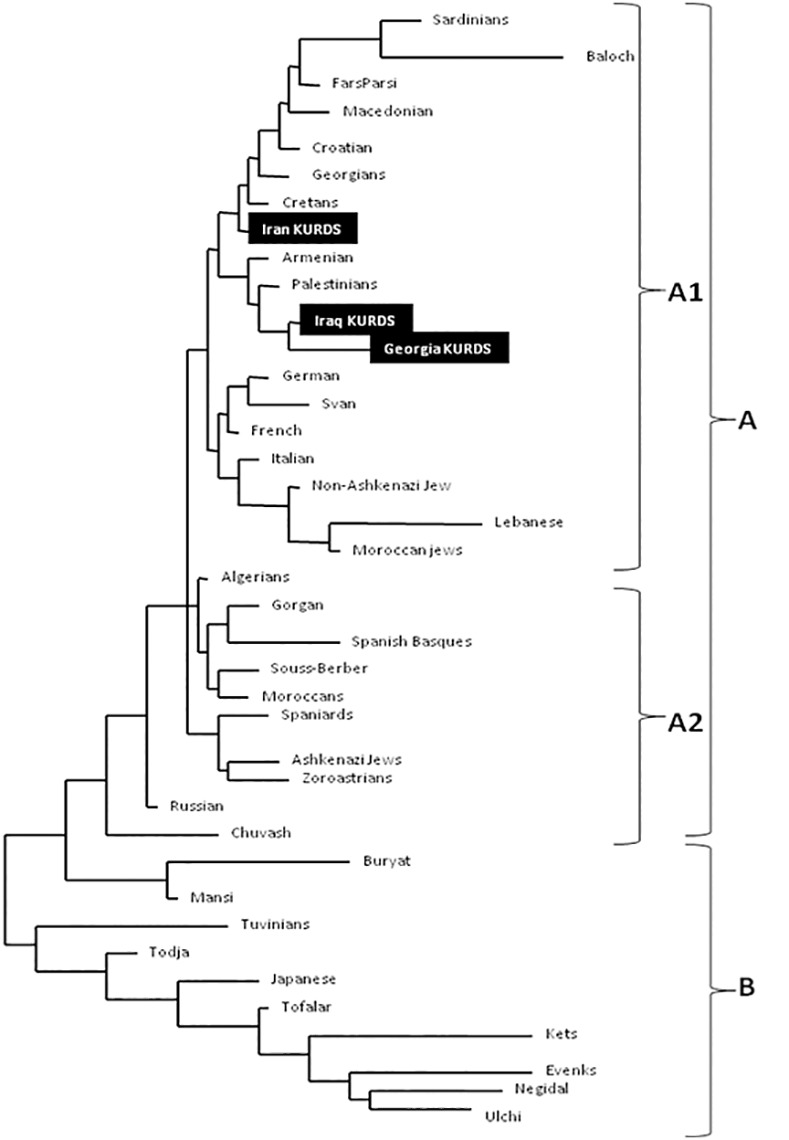
Neighbour-Joining dendrogram. Neighbour-Joining (NJ) dendrogram constructed with HLA-DRB1 allele frequencies showing relatedness between Iraq Kurds and other World populations. Bootstrap values are 100%.

NJ relatedness dendrogram based on HLA-DRB1 analysis separates populations in two differentiated clusters: A and B (Fig 2). Cluster A groups North and South Mediterraneans (Europeans and Africans) and Middle East populations (included Iraq Kurds, Iran Kurds [13] and Georgia Kurds [5]). Cluster B includes Central and eastern Siberians and Oriental population: such Tuvinians [3], Todja, Tofalar, Ulchi, Negidal [31], Japanese [20], Kets, Evenks [34]. Cluster A1 contains Iraq Kurds and Georgia Kurds, which are placed together [5], Palestinians [41], Armenians [20], Iran Kurds [13], East Europe (Croatians) [21], Cretans [33], Macedonians [38], Near East (Lebanese) [21] and Non-Ashkenazi Jews [28] (Fig 2). Cluster A2 places together other Mediterraneans (Europeans and Africans such as Spaniards and Spanish Basques) [43], French [20], Berbers [30] and Moroccans [39] (Fig 2).

**Fig 3:**
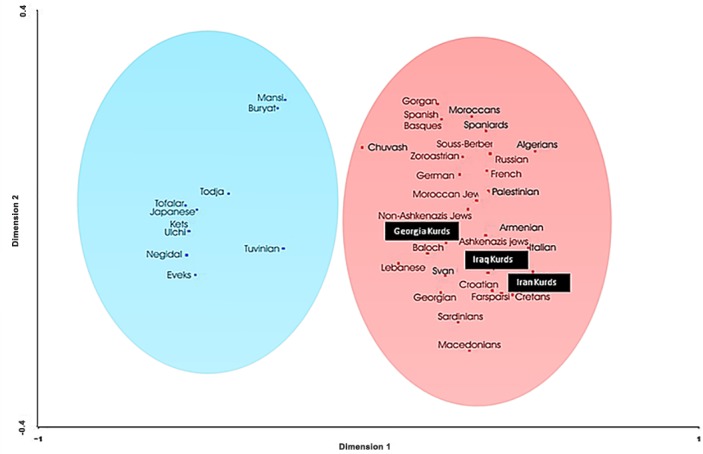
Correspondence analysis. Correspondence analysis showing a global view of the relationship between Kurds and Mediterranean, Siberians and other World populations according to HLA-DRB1 (low resolutions) allele frequencies in three dimensions (bidimensional representation).

Correspondence analysis based on HLA-DRB1 allele frequencies (Fig 3) shows similar results to those of Fig 2. Two clusters are clearly defined according to first dimension that explains most of the variability among populations. The first one groups together Siberian and Oriental populations (left, Fig 3) and the second cluster comprises Europeans, Mediterraneans, Caucasus and Iranian populations; Iraq Kurds, Iran Kurds [13] and Georgia Kurds [5] are located relatively close together.

Plain genetic distances (DA) show that Iraq Kurds closest genetic distances are the following: Near East populations (Iran Kurds, Palestinians, FarsParsi, Georgia Kurds and Ashkenazi Jews), eastern Mediterranean populations (Armenians, Cretans and Macedonians), and Mediterranean populations (Sardinians, Spaniards, Algerians and Italians) (Results not shown).

### HLA-A, -B. DRB1 and -DQB1 extended haplotype analysis in Kurds: comparison with other populations

Associations between different HLA *loci* were estimated in Iraq Kurds (Table 4). The most frequent five *loci* haplotype (A-B-C-DRB1-DQB1) were obtained. Eight Mediterranean five *loci* haplotype are found (A*03-B*44-C*16-DR*04-DQ*03, A*26-B*08-C*07-DR*03-DQ*02, A*33-B*14-C*08-DR*01-DQ*05, A*01-B*52-C*12-DR*15-DQ*06, A*01-B*08-C*07-DR*03-DQ*02, A*02-B*44-C*05-DR*11-DQ*03, A*01-B*35-C*04-DR*14-DQ*05 and A*02-B*51-C*16-DR*15-DQ*06) and three Near East five *loci* haplotypes (A*03-B*35-C*04-DR*11-DQ*03, A*02-B*51-C*15-DR*11-DQ*03 and A*02-B*51-C*14-DR*11-DQ*03) represents about 4.01% (Table 4 and footnote). Also, an Eurasiatic haplotype is found: A*24-B*35-C*04-DR*11-DQ*03. These results show HLA genetic characteristics of both Mediterranean and Near East populations; also one Eurasiatic haplotype is found in a relatively high frequency. References for Table 4 footnote are: *a* [45], *b* [32, 36, 41], *c* [45], *d [35],* e [32, 33, 39, 41, 46], *f* [38], *g* [32, 33, 38, 41, 47, 48, 49], *h* [50], *i* [35], *k* [35], *j* [45] and *l* [51].

**Table 4:** The twelve most frequent HLA-A, -B, -C, -DRB1 and -DQB1 extended haplotypes in Kurds.

Haplotype	HF (%)	Possible Origin
A*03-B*44-C*16-DR*04-DQ*03^*a*^	3.30	Mediterranean
A*24-B*35-C*04-DR*11-DQ*03^*b*^	2.59	Euroasiatic
A*26-B*08-C*07-DR*03-DQ*02^*c*^	2.39	Mediterranean
A*03-B*35-C*04-DR*11-DQ*03^*d*^	1.71	Near East
A*33-B*14-C*08-DR*01-DQ*05^*e*^	1.67	Mediterranean
A*01-B*52-C*12-DR*15-DQ*06^*f*^	1.59	Mediterranean
A*01-B*08-C*07-DR*03-DQ*02^*g*^	1.44	Mediterranean
A*02-B*44-C*05-DR*11-DQ*03^*h*^	1.20	Mediterranean
A*02-B*51-C*15-DR*11-DQ*03^*i*^	1.20	Near East
A*01-B*35-C*04-DR*14-DQ*05^*j*^	1.17	Mediterranean
A*02-B*51-C*14-DR*11-DQ*03^*k*^	1.10	Near East
A*02-B*51-C*16-DR*15-DQ*06^*l*^	1.05	Mediterranean

### HLA haplotype common to georgian, Iran and Iraq Kurds

The following haplotypes are shared among Kurd populations: HLA-B*52-DRB1*15 is present in Iran Kurds (2.5%), Iraq Kurds (1.59%) and Georgia Kurds (3.6%); these later results have been taken from our previous work with a low number of individuals (n = 30) [5]. HLA-A*02-B*51-DRB1*11 is present in Iraq Kurds (2.3%) and Georgia Kurds (3.6%) (this study and [5,13]); DRB1*11-DQB1*03 is present in Iraq Kurds (10%) and Iran Kurds (6.7%); DRB1*03-DQB1*02 is present in Iraq Kurds (5.9%) and Iran Kurds (3.38%); DRB1*01-DQB1*05 is present in Iraq Kurds (3.3%) and Iran Kurds (1.67%) and DRB1*15-DQB1*06 is present in Iraq Kurds (2.5%) and in Iran Kurds (1.59%) (this study and [5,13]).

## Conclusions and Final Remarks

Kurds are currently living in Kurdistan, a region encompassing different parts of several Middle East countries (Fig 1, Table 1); in addition they have moved to live in Middle East and European cities. We had previously studied Kurds in Tbilisi (Georgia, [5]) and also in Iran [13] for HLA allele frequencies. Our conclusions were that their HLA profile showed that Kurds form part of Mediterranean stock of people and also had Caucasus genetic traits (Svan, Georgians, [13,35,44]). Also, it is worth mentioning that Lak population (East Caucasus Area) may be close to both Lur and Kurd populations, and Lak name could be considered composed of Lur and Kurd words [52].

In the present paper, we have analyzed HLA genes in Kurds living in North Iraq (Erbil and Dohuk areas). Comparison with other populations place them as a Middle East population, close to Kurds from Tbilisi-Georgia, Palestinians, Armenians and Kurds from Iran (Fig 2). Correspondence analysis (Fig 3, right side) shows that Kurds (living in Iran, Tbilisi-Georgia and Iraq) somewhat divide bidimensional representation analysis in western Mediterraneans (upper part) and Eastern Mediterraneans (lower part). In both analyses (Figs 2 and 3), Kurds are also close to Caucasian (Svan, Georgian) populations. Conclusion is that Kurds are genetically close to surrounding Caucasian and Mediterranean populations and that have remained settled down in Kurdistan since ancient times; supporting historical evidence is reviewed in our previous work [5,6] and Ref [15].

HLA genetic similarity have been reported between Turks (whose genes belong to old Anatolian stock) and Kurds [5,6,13]. Kurds and Turks speak languages that are included in different families [53]. However, Kurd HLA genetic studies include them into Mediterranean stock together with Turks [5,13]. Other genetic studies based on Y-Chr in Kurds from Turkey, Georgia and Iran identify the dominant presence of haplogroups originated in Middle East (Anatolia or Mesopotamia) that show a close association with Jews, Lebanese and Turkish genes [7,8,12]. Also, Iranian populations are close to Kurds [54,55]. This again shows that languages and genes do not correlate because languages may be imposed by a genetic (but powerful) minority. This is the case of Turks: Anatolian people were settled down there since ancient prehistoric times, but a minority of people (Turks) coming from Central Asia imposed language in historical times [5,6].

Thus Middle East peoples from Mediterranean border and Kurds seem originally to belong to a similar ethnic group according to HLA autosomic and Y chromosomes genes results. Kurds have always lived in the mountains being "autochthonous” (6000 BC). Hurrians, whose language was Caucasian (and not Indo-European) may be Kurds ancient genetic background, reviewed in Refs [5,6]. By 1200 BC, Medes and others invaded Hurrian area. Kurdish historians consider that Kurds come from Medes, reviewed in [5]. "*Kuru*" was the first name of Kurds given by Assyrians (1000 BC) to groups living at Mt. Azu, Kurdistan. Kurds are also mentioned by early classical historians like Polybius (133 BC) and Strabo (48 AD), Kurds were named as “the Mountains People” under Persian, Greek and Roman Anatolian Peninsula rule [5].

In summary, all three Kurd populations studied in the present paper are genetically close together and to other Mediterranean and Caucasus populations according to HLA genes. This study may also help for future transplantation programs in their area and Kurd HLA Epidemiology and Pharmacogenomics.
